# Supplementary figures and images for: Global analysis of N6-methyladenosine functions and its disease association using deep learning and network-based methods
Source: PLoS Comput Biol. 2019 Jan 2;15(1):e1006663. doi: 10.1371/journal.pcbi.1006663 (PMC6331136; doi:10.1371/journal.pcbi.1006663)

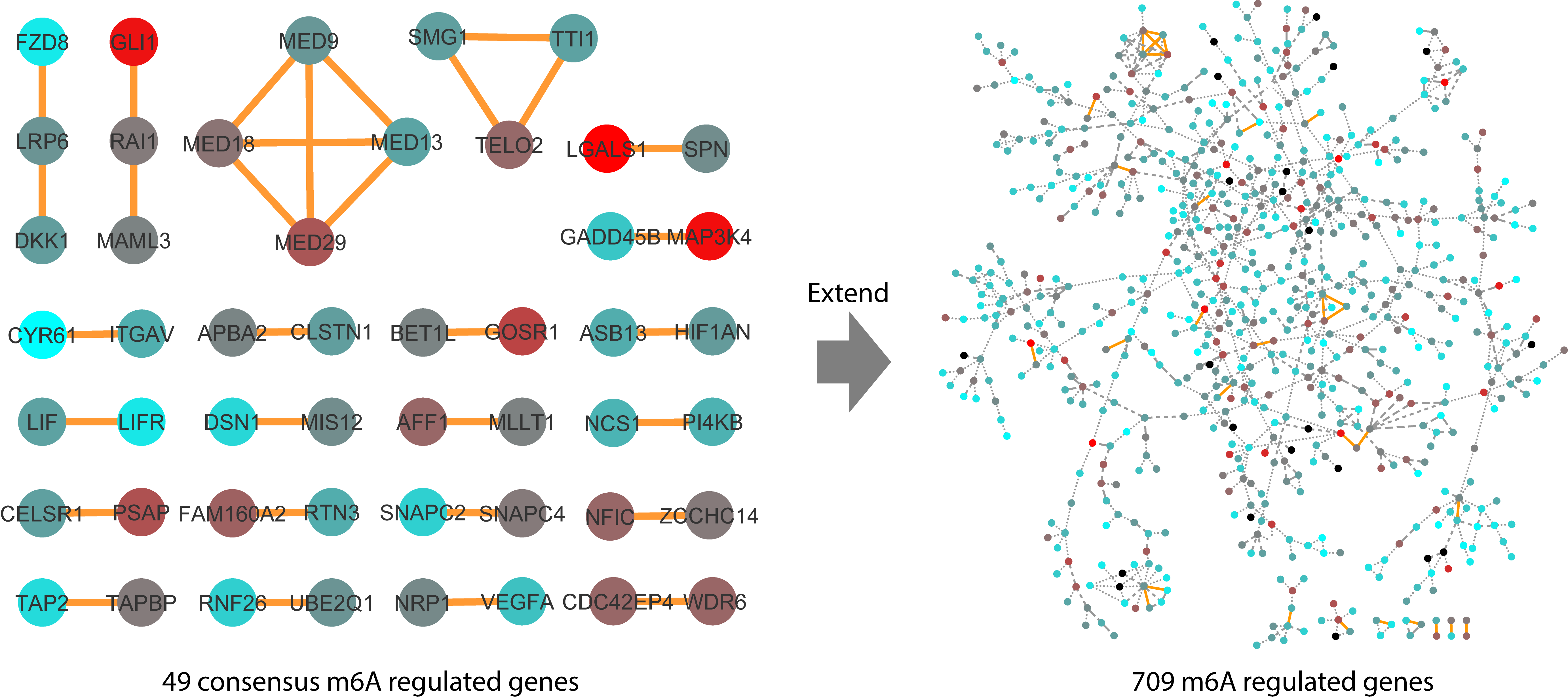

Supplement: S1 Fig — The color of the node denotes the heat i.e. expression-methylation correlation degree it has, red denotes higher and blue denotes lower. The solid orange edge is edge that identified in all 4 PPI networks, the dashed edge is edge that identified in at least 3 networks, the dash dot edge is in 2 networks and the dotted is 1. (TIF) [file pcbi.1006663.s001.tif]

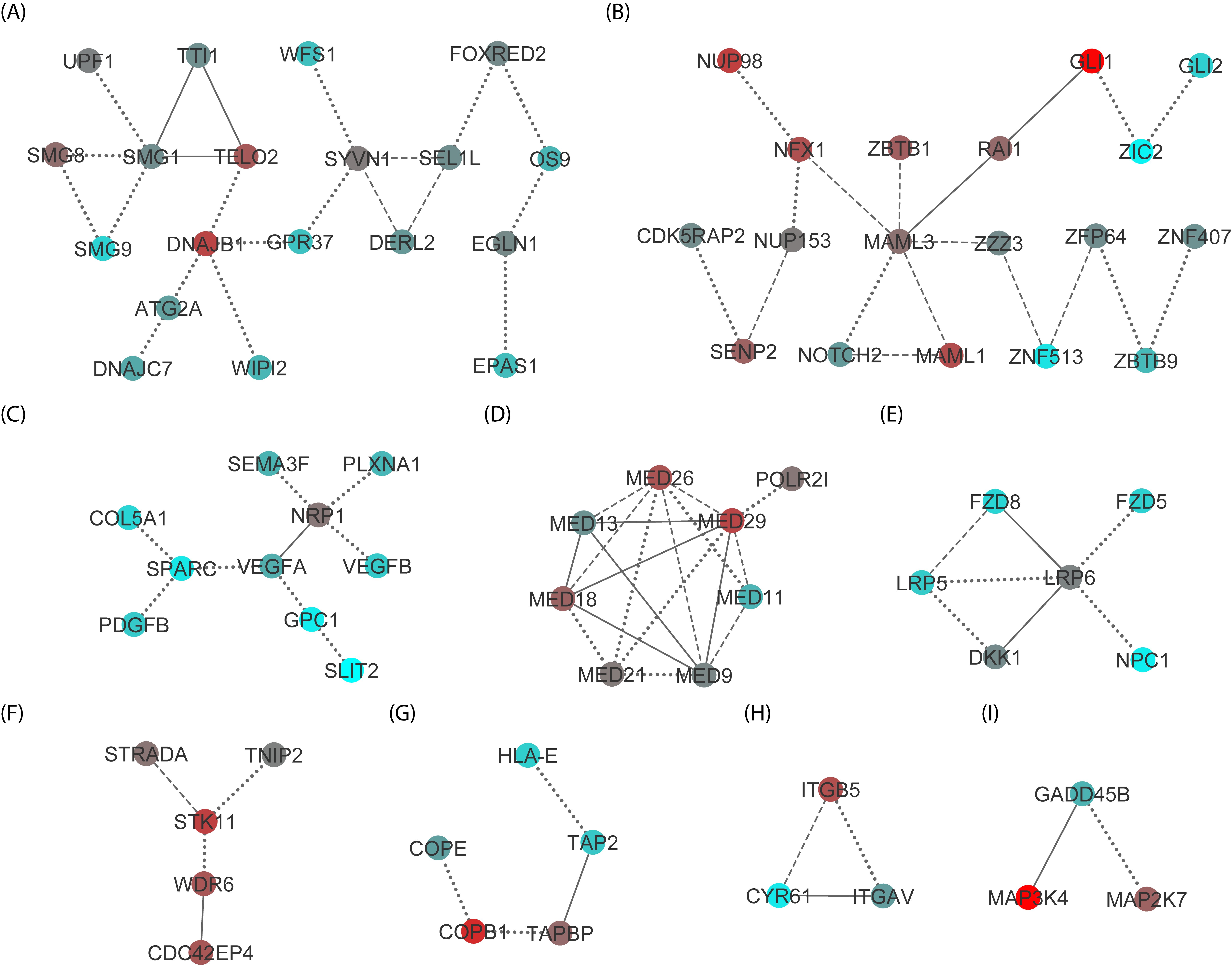

Supplement: S2 Fig — (A)-(I) denotes the 9 functional significant subnetworks of m6A-regulated genes. The color of the node denotes the heat i.e. expression-methylation correlation degree it has, red denotes higher and blue denotes lower. The solid edge is edge that identified in all 4 PPI networks, the dashed edge is edge that identified in at least 3 networks and the dotted edge is in 2 networks. (TIF) [file pcbi.1006663.s002.tif]

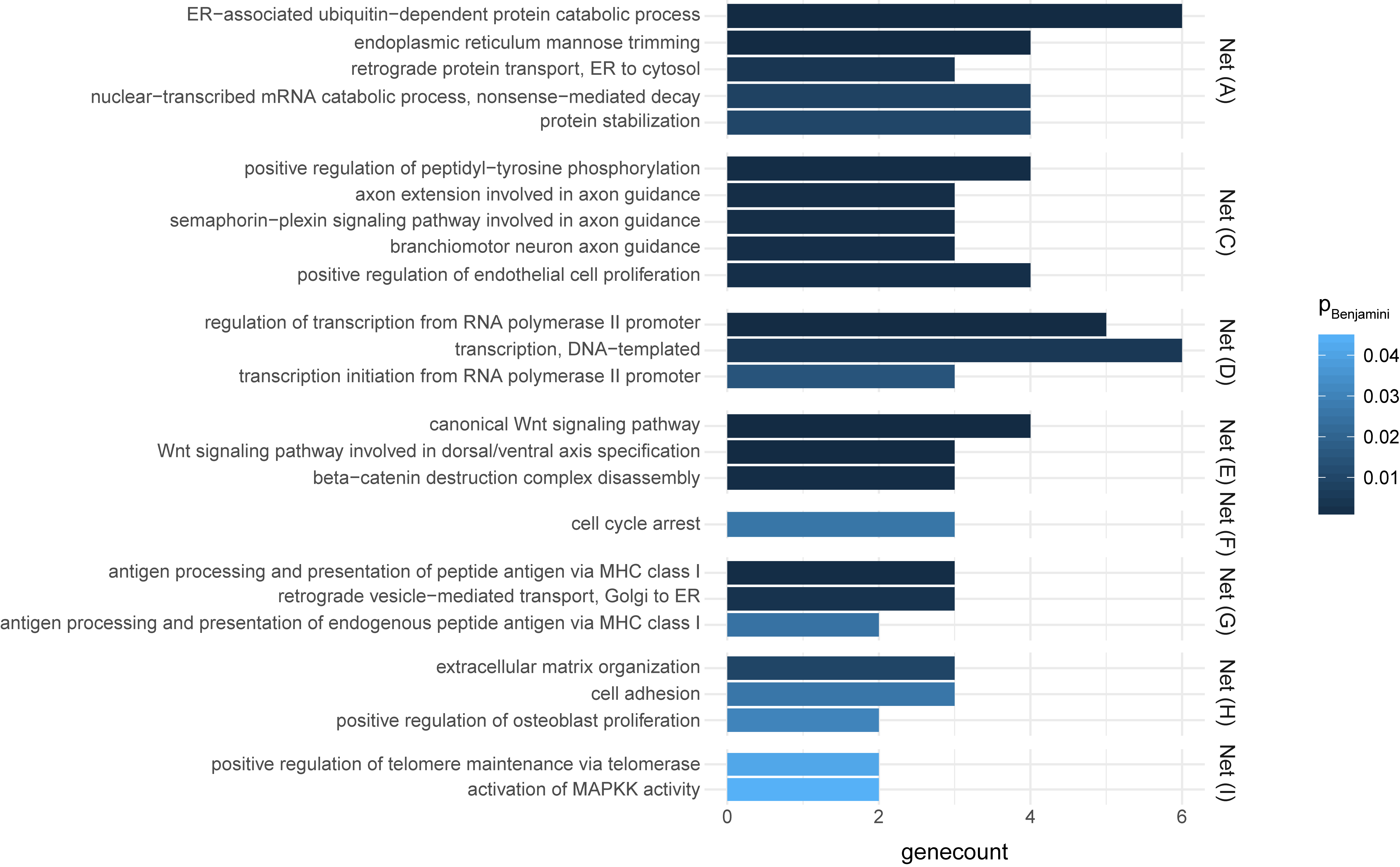

Supplement: S3 Fig — There is no significant BP for subnetwork (B). Gene count means number of genes involved in the corresponding BP terms and PBenjamini is the adjusted FDR of enriched p-value. All the involved BP terms have a PBenjamini < 0.05 and we only list the top 5 if the enriched terms are more than 5. (TIF) [file pcbi.1006663.s003.tif]

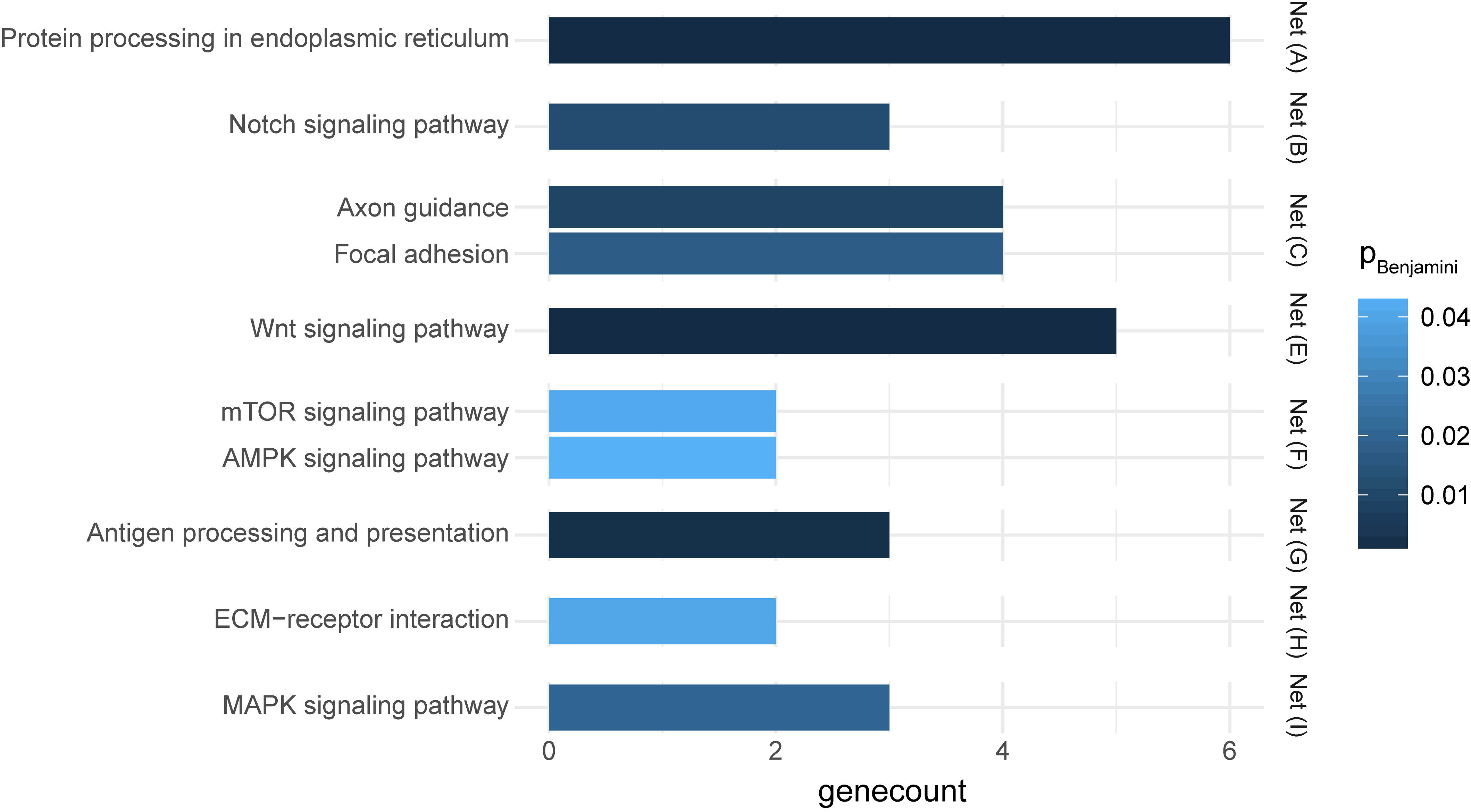

Supplement: S4 Fig — There is no significant KEGG pathways for network (D). Gene count means number of genes involved in the corresponding pathways and PBenjamini is the adjusted FDR of enriched p-value. All the involved pathways have a PBenjamini < 0.05. (TIF) [file pcbi.1006663.s004.tif]

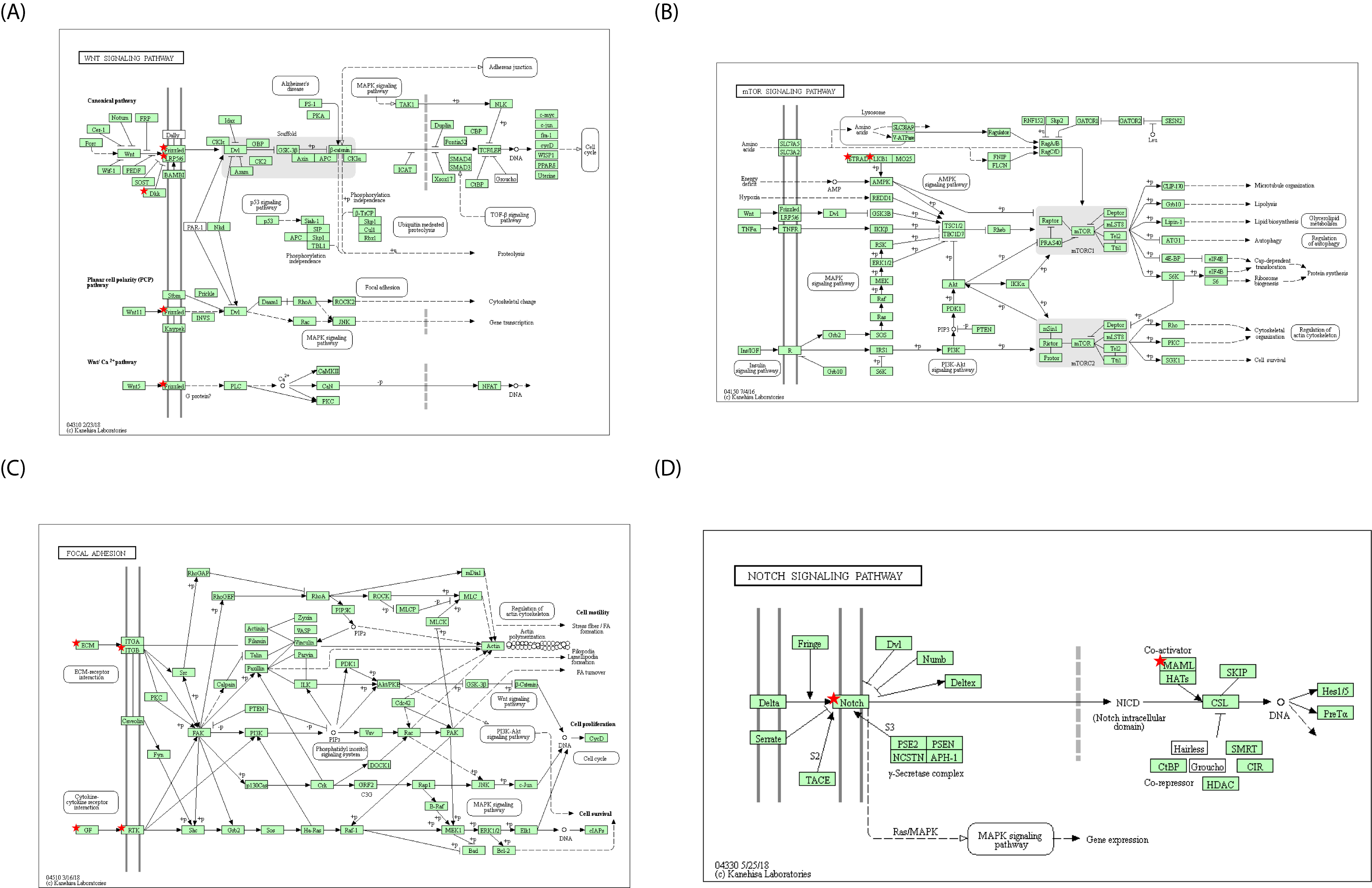

Supplement: S5 Fig — (A) is WNT signaling pathway. (B) is mTOR signaling pathway. (C) is Focal adhesion. (D) is Notch signaling pathway. The genes marked with red star are m6A regulated genes in the subnetworks. As is shown, m6A regulated genes tend to be in the upstream of these enriched pathways. (TIF) [file pcbi.1006663.s005.tif]

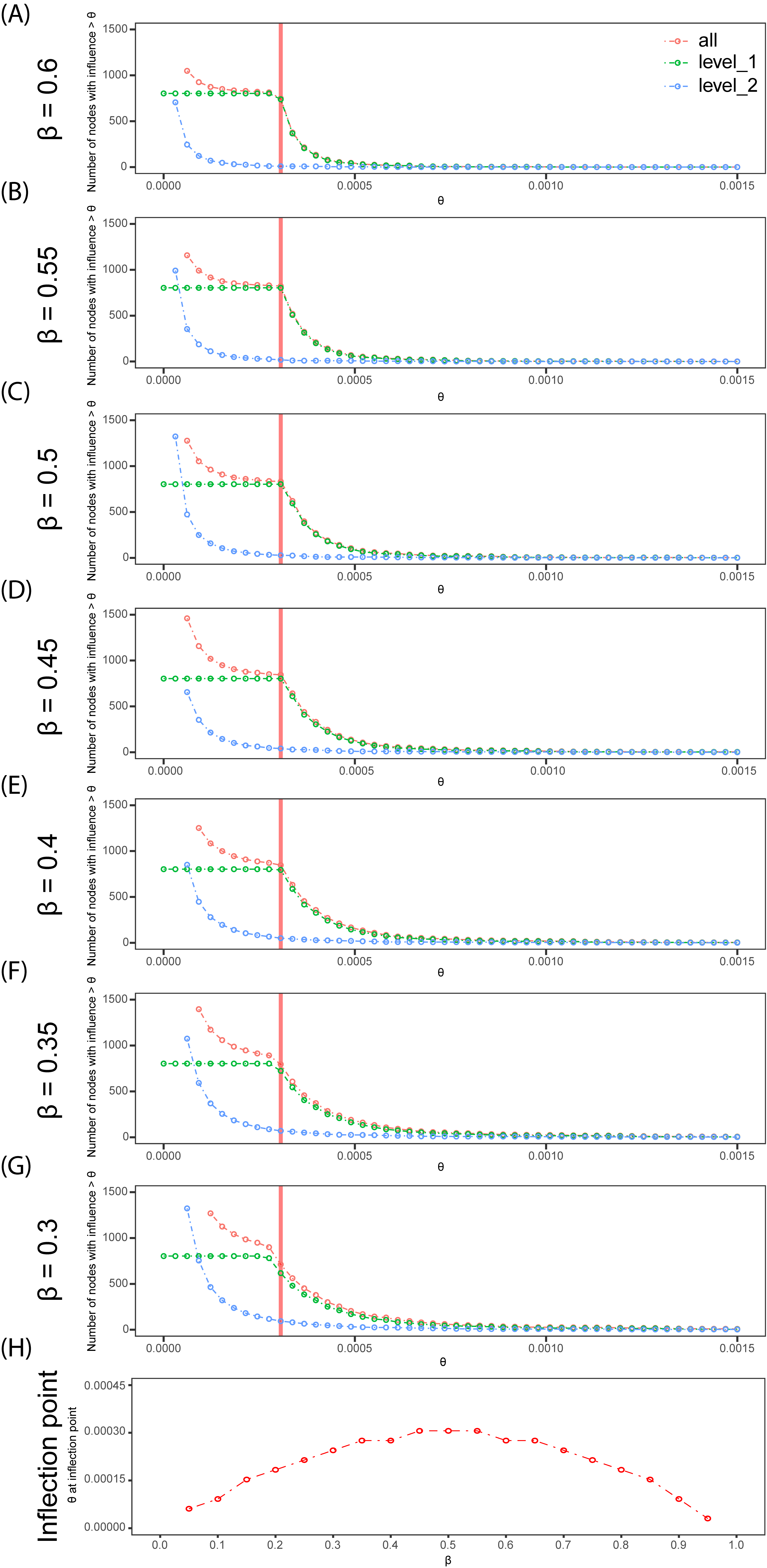

Supplement: S6 Fig — Figures (A)-(G) represent the distributions of number of nodes with influence larger than a cutoff for β from 0.6 to 0.3 for gene TP53, which has a high betweenness centrality. The x-axis of each distribution represents θ, a cutoff of influence. The y-axis represents the number of nodes in the interaction network with influence larger than θ. Red dotted circles denote the number of all nodes with an influence larger than different θs, green dotted circles denote that of the level-one nodes, and blue dotted circles denote that of the level-two nodes. The red vertical lines in all the distributions represent the location of the inflection point in level one for the β we chose for different interaction networks, β = 0.5 for BioGRID. (H) depicts the inflection point θ as a function of β ranging from 0.05 to 0.95. (TIF) [file pcbi.1006663.s006.tif]
